# Tracking the Turn Maneuvering Target Using the Multi-Target Bayes Filter with an Adaptive Estimation of Turn Rate

**DOI:** 10.3390/s17020373

**Published:** 2017-02-15

**Authors:** Zong-xiang Liu, De-hui Wu, Wei-xin Xie, Liang-qun Li

**Affiliations:** College of Information Engineering, Shenzhen University, Shenzhen 518060, China; 2151130216@email.szu.edu.cn (D.W.); wxxie@szu.edu.cn (W.X.); lqli@szu.edu.cn (L.L.)

**Keywords:** target tracking, Bayes filter, maneuvering target, estimation of turn rate, multiple models

## Abstract

Tracking the target that maneuvers at a variable turn rate is a challenging problem. The traditional solution for this problem is the use of the switching multiple models technique, which includes several dynamic models with different turn rates for matching the motion mode of the target at each point in time. However, the actual motion mode of a target at any time may be different from all of the dynamic models, because these models are usually limited. To address this problem, we establish a formula for estimating the turn rate of a maneuvering target. By applying the estimation method of the turn rate to the multi-target Bayes (MB) filter, we develop a MB filter with an adaptive estimation of the turn rate, in order to track multiple maneuvering targets. Simulation results indicate that the MB filter with an adaptive estimation of the turn rate, is better than the existing filter at tracking the target that maneuvers at a variable turn rate.

## 1. Introduction

Target tracking has been discussed in many articles due to its military and civil applications, which range from threat warnings, to intelligent surveillance and situational awareness [1,2,3,4,5,6,7,8,9]. Maneuvering target tracking is the most essential ingredient of target tracking and has attracted the attention of many researchers. A number of efficient tracking algorithms for maneuvering targets have been developed and designed in the past few decades [10,11,12,13,14,15,16,17,18]. The interacting multiple model (IMM) algorithms were independently developed in [10,11], in order to track the maneuvering target in systems with Markov-switching coefficients, and in air traffic control, respectively. The mode-set adaptive IMM algorithm and multiple model method with variable structure were designed in [12,13], to improve the performance of IMM algorithms when tracking a maneuvering target. By combining the IMM method with the joint probabilistic data association (JPDA) and multiple hypothesis tracking (MHT) techniques, respectively, the IMM-JPDA filter and the IMM-MHT filter were developed in [14,15], in order to track multiple maneuvering targets. By applying the switching multiple models technique to the probability hypothesis density (PHD) filter and multi-target Bayes (MB) filter, respectively, Pasha developed a PHD filter to track maneuvering targets in the presence of clutter and noise [16], and Liu designed a MB filter for multiple maneuvering target tracking, in the case of low detection probability [17].

As mentioned above, the existing methods for maneuvering target tracking apply the IMM approach, or the switching multiple models technique, to the tracking filter. In these methods, a finite set of dynamic models are used each time. Because the motion mode space of a target is continuous, a sufficiently large set of dynamic models is usually required to cover the range of possible motion modes of the target. Such a large set is impractical because an increase in the number of dynamic models, also leads to an increase in the computational load. Additionally, it is worth noting that the actual motion mode of a target at any given time, may be different from all of the dynamic models, even if a sufficiently large set of dynamic models are used by the filter.

When tracking the target that maneuvers at a variable turn rate, a limited set of dynamic models with different turn rates are usually used by the filter [12,16]. Since the turn rate of a target at any given time is unknown and random, its actual turn rate at any given time may be different from the turn rates in dynamic models. This difference causes the filter to provide an inaccurate state estimation of the target at this time. 

To track the target that maneuvers at a random turn rate, we establish a formula for computing the turn rate of the target. This formula solves the estimation issue of the turn rate by using the state vector of a target at a previous time, and its measurement at the current time. Applying the estimation method of the turn rate to the MB filter, we present the MB filter with an adaptive estimation of the turn rate. Its performance is demonstrated by the simulation results.

## 2. A Brief Description of Pasha’s PHD Filter

Pasha’s Gaussian mixture PHD filter is applied to the tracking of multiple maneuvering targets in systems with linear Gaussian jump Markov system models, and is used as the comparison object in this paper. We will first give a brief description of this filter. A simplified version of Pasha’s Gaussian mixture PHD filter is composed of the following four steps:

### Step 1: Prediction

Let $v_{k-1}(x_{k-1},r_{k-1})=\sum_{i=1}^{N_{k-1}}w_{i,k-1}(r_{i,k-1})N(x_{i,k-1};m_{i,k-1}(r_{i,k-1}),P_{i,k-1}(r_{i,k-1}))$ denote the posterior intensity at time k−1, where $N_{k-1}$ is the number of Gaussian terms at time k−1 and N(⋅;m,P) is a Gaussian distribution with mean vector m, and covariance matrix P; $r_{i,k-1}$, $x_{i,k-1}$, $w_{i,k-1}(r_{i,k-1})$, $m_{i,k-1}(r_{i,k-1})$, and $P_{i,k-1}(r_{i,k-1})$ are the model label, state vector, weight, mean vector, and covariance matrix of Gaussian term i, respectively. The predicted posterior intensity is given by:

(1) $$v_{k|k-1}(x_{k},r_{k})=\sum_{i=1}^{N_{k-1}}\sum_{r_{i,k}=1}^{M_{r}}w_{i,k|k-1}(r_{i,k})N(x_{i,k};m_{i,k|k-1}(r_{i,k}),P_{i,k|k-1}(r_{i,k}))$$

where $M_{r}$ is the number of models used, and $w_{i,k|k-1}(r_{i,k})$, $m_{i,k|k-1}(r_{i,k})$, and $P_{i,k|k-1}(r_{i,k})$ are given by:

(2) $$w_{i,k|k-1}(r_{i,k})=p_{S,k}t_{k|k-1}(r_{i,k}|r_{i,k-1})w_{i,k-1}(r_{i,k-1})$$

(3) $$m_{i,k|k-1}(r_{i,k})=F_{k-1}(r_{i,k})m_{i,k-1}(r_{i,k-1})$$

(4) $$P_{i,k|k-1}(r_{i,k})=Q_{k-1}(r_{i,k})+F_{k-1}(r_{i,k})P_{i,k-1}(r_{i,k-1})F_{k-1}^{T}(r_{i,k})$$

where $p_{S,k}$ is the survival probability; $F_{k-1}(r_{i,k})$ and $Q_{k-1}(r_{i,k})$ are the state transition and process noise covariance matrices, respectively, of model $r_{i,k}$; and $t_{k|k-1}(r_{i,k}|r_{i,k-1})$ is the Markov transition probability from model $r_{i,k-1}$ to model $r_{i,k}$.

### Step 2: Update

If the predicted posterior intensity is given by Equation (1), then the updated posterior intensity is given by:

(5) $$\begin{array}{cc} v_{k|k}(x_{k},r_{k})= & \sum_{i=1}^{N_{k-1}}\sum_{r_{i,k}=1}^{M_{r}}w_{i,k|k}(r_{i,k})N(x_{i,k};m_{i,k|k-1}(r_{i,k}),P_{i,k|k-1}(r_{i,k}))\\ & +\sum_{j=1}^{M_{k}}\sum_{i=1}^{N_{k-1}}\sum_{r_{i,k}}^{M_{r}}w_{i,k|k}^{j}(r_{i,k})N(x_{i,k};m_{i,k|k}^{j}(r_{i,k}),P_{i,k|k}^{j}(r_{i,k})) \end{array}$$

where:

(6) $$w_{i,k|k}(r_{i,k})=(1-p_{D,k})w_{i,k|k-1}(r_{i,k})$$

(7) $$m_{i,k|k}^{j}(r_{i,k})=m_{i,k|k-1}(r_{i,k})+A_{i,k}(r_{i,k})\cdot (y_{j,k}-H(r_{i,k})m_{i,k|k-1}(r_{i,k}))$$

(8) $$P_{i,k|k}^{j}(r_{i,k})=(I-A_{i,k}(r_{i,k})\cdot H(r_{i,k}))P_{i,k|k-1}(r_{i,k})$$

(9) $$A_{i,k}(r_{i,k})=P_{i,k|k-1}(r_{i,k})H^{T}(r_{i,k}){\left[H(r_{i,k})P_{i,k|k-1}(r_{i,k})H^{T}(r_{i,k})+R(r_{i,k})\right]}^{-1}$$

(10) $$w_{i,k|k}^{j}(r_{i,k})=\frac{p_{D,k}w_{i,k|k-1}(r_{i,k})N(y_{j,k};H(r_{i,k})m_{i,k|k-1}(r_{i,k}),H(r_{i,k})P_{i,k|k-1}(r_{i,k})H^{T}(r_{i,k})+R(r_{i,k}))}{\lambda_{c,k}+p_{D,k}\sum_{e=1}^{N_{k-1}}\sum_{r_{e,k}=1}^{M_{r}}w_{e,k|k-1}(r_{e,k})N(y_{j,k};H(r_{e,k})m_{e,k|k-1}(r_{e,k}),H(r_{e,k})P_{e,k|k-1}(r_{i,k})H^{T}(r_{e,k})+R(r_{e,k}))}$$

where $M_{r}$ is the number of measurements at time k, $y_{j,k}$ denotes a measurement at time k; $H(r_{i,k})$ and $R(r_{i,k})$ are the observation matrix and covariance matrix of observation noise, respectively; and I, $\lambda_{c,k}$, and $p_{D,k}$ denote the identity matrix, clutter rate, and detection probability, respectively.

### Step 3: Generation of the Birth Intensity

The birth intensity is generated from the measurements at time k and is given by:

(11) $$\gamma (x_{k},r_{k})=\sum_{j=1}^{M_{k}}w_{\gamma ,k}^{j}N(x_{j,k};m_{\gamma ,k}^{j}(r_{j,k}),P_{\gamma ,k}^{j}(r_{j,k}))$$

where $m_{\gamma ,k}^{j}$ is taken from measurement $y_{j,k}={\left[\begin{array}{cc} r_{x,k}^{j} & r_{y,k}^{j} \end{array}\right]}^{T}$; and $m_{\gamma ,k}^{j}={\left[\begin{array}{cccc} r_{x,k}^{j} & 0 & r_{y,k}^{j} & 0 \end{array}\right]}^{T}$, $r_{j,k}=1$, $w_{\gamma ,k}^{j}=\rho_{r}$, and $P_{\gamma ,k}^{j}=P_{\gamma }$ where $\rho_{r}$ and $P_{\gamma }$ are the known parameter and covariance matrix, respectively.

### Step 4: Combination of the Updated Posterior Intensity and Birth Intensity

The posterior intensity at time k is obtained by the combination of the updated posterior intensity in Equation (5), and the birth intensity in Equation (11), which is given by:

(12) $$\begin{array}{cc} v_{k}(x_{k},r_{k}) & =v_{k|k}(x_{k},r_{k})+\gamma (x_{k},r_{k})\\ & =\sum_{i=1}^{N_{k-1}}\sum_{r_{i,k}=1}^{M_{r}}w_{i,k|k}(r_{i,k})N(x_{i,k};m_{i,k|k-1}(r_{i,k}),P_{i,k|k-1}(r_{i,k}))\\ & +\sum_{j=1}^{M_{k}}\sum_{i=1}^{N_{k-1}}\sum_{r_{i,k}}^{M_{r}}w_{i,k|k}^{j}(r_{i,k})N(x_{i,k};m_{i,k|k}^{j}(r_{i,k}),P_{i,k|k}^{j}(r_{i,k}))+\sum_{j=1}^{M_{k}}w_{\gamma ,k}^{j}N(x_{j,k};m_{\gamma ,k}^{j},P_{\gamma ,k}^{j}) \end{array}$$

After this combination, the Gaussian terms whose weight is less than threshold τ are pruned, and the posterior intensity, which is composed of the remaining Gaussian terms, is propagated to the next time step. Those Gaussian terms whose weight is greater than 0.5 are picked as the output of the filter at time k.

## 3. Estimation of Turn Rate

**Figure 1:**
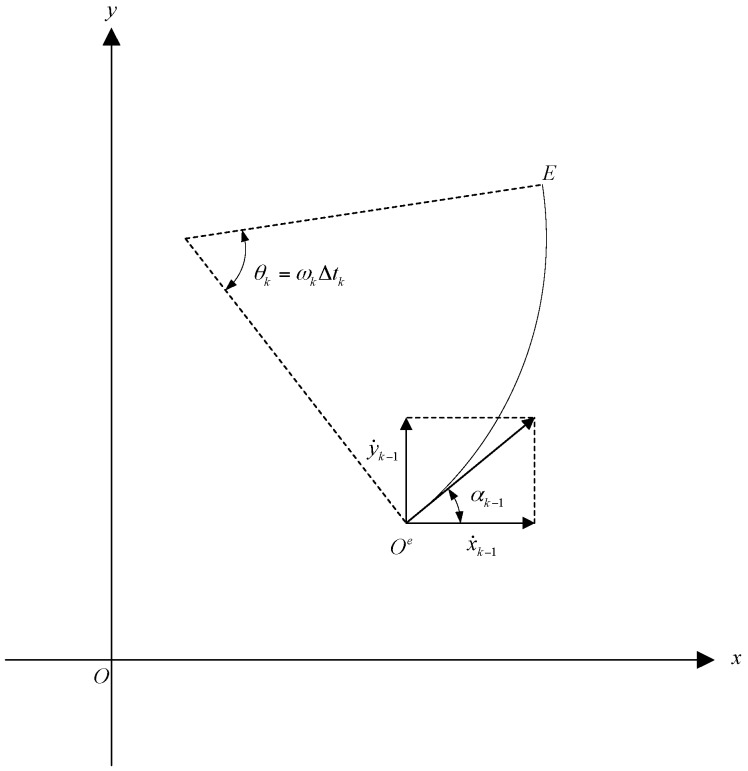
Moving trajectory for a target with turn rate $\omega_{k}$.

In this Section, we will estimate the turn rate of a maneuvering target by using its state vector at time k−1 and its position measurement at time k. Figure 1 shows a maneuvering target with turn rate $\omega_{k}$ which moves from point $O^{e}$ at time k−1, to point E at time k. Let $X_{k-1}={\left[\begin{array}{cccc} x_{k-1} & {\dot{x}}_{k-1} & y_{k-1} & {\dot{y}}_{k-1} \end{array}\right]}^{T}$ and $X_{k}={\left[\begin{array}{cccc} x_{k} & {\dot{x}}_{k} & y_{k} & {\dot{y}}_{k} \end{array}\right]}^{T}$ denote the state vectors of the target at times k−1 and k, respectively, where $\left(x_{k-1},y_{k-1}\right)$ and $\left(x_{k},y_{k}\right)$ denote the position coordinates of the target at times k−1 and k, respectively, and $\left({\dot{x}}_{k-1},{\dot{y}}_{k-1}\right)$ and $\left({\dot{x}}_{k},{\dot{y}}_{k}\right)$ denote its velocities at times k−1 and k, respectively. Obviously, in the x-y Cartesian coordinate system, the Cartesian coordinates of points $O^{e}$ and E are $\left(x_{k-1},y_{k-1}\right)$ and $\left(x_{k},y_{k}\right)$, respectively.

**Figure 2:**
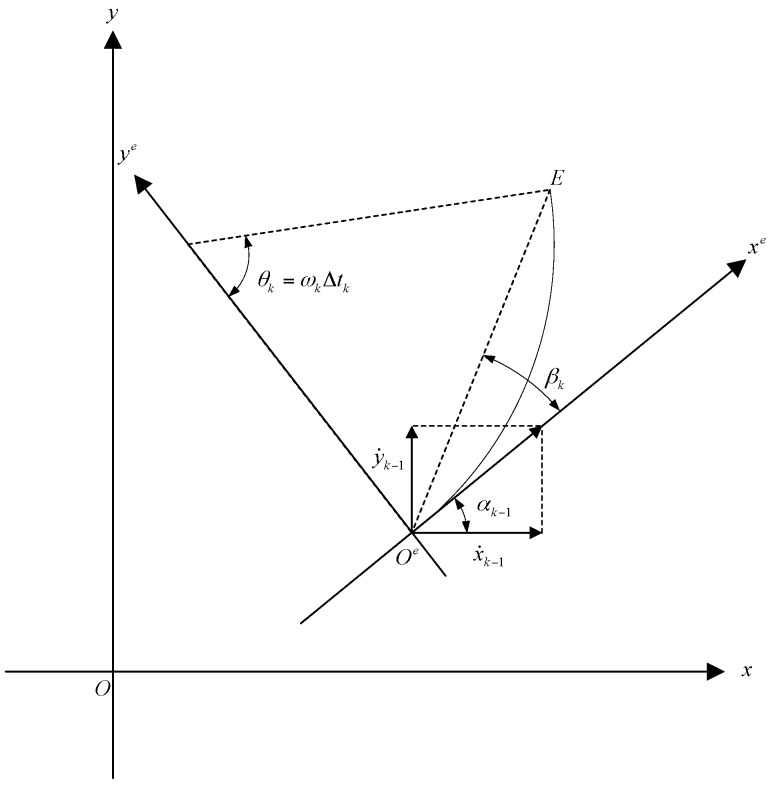
Transformation between two coordinate systems.

We introduce an $x^{e}$ − $y^{e}$ Cartesian coordinate system, whose origin is located at point $O^{e}$. The introduced $x^{e}$ − $y^{e}$ Cartesian coordinate system is shown in Figure 2. The coordinate transformation from position coordinates X in the x-y coordinate system, to position coordinates $X^{c}$ in the $x^{e}$ − $y^{e}$ coordinate system, is given by:

(13) $$X^{c}=\left[\begin{array}{cc} \cos\alpha_{k-1} & \sin\alpha_{k-1}\\ -\sin\alpha_{k-1} & \cos\alpha_{k-1} \end{array}\right]\times \left(X-\left[\begin{array}{c} x_{k-1}\\ y_{k-1} \end{array}\right]\right)$$

where $\alpha_{k-1}$ is given by:

(14) $$\alpha_{k-1}=arc\cos\frac{{\dot{x}}_{k-1}}{\sqrt{{\dot{x}}_{k-1}^{2}+{\dot{y}}_{k-1}^{2}}}$$

Similarly, the vector transformation from velocity vector V in the x − y coordinate system, to velocity vector $V^{c}$ in the $x^{e}$ − $y^{e}$ coordinate system, is given by:

(15) $$V^{c}=\left[\begin{array}{cc} \cos\alpha_{k-1} & \sin\alpha_{k-1}\\ -\sin\alpha_{k-1} & \cos\alpha_{k-1} \end{array}\right]\times V$$

Using Equations (13) and (15) to address position coordinates $X={\left[\begin{array}{cc} x_{k-1} & y_{k-1} \end{array}\right]}^{T}$ and velocity vector $V={\left[\begin{array}{cc} {\dot{x}}_{k-1} & {\dot{y}}_{k-1} \end{array}\right]}^{T}$ in state vector $X_{k-1}={\left[\begin{array}{cccc} x_{k-1} & {\dot{x}}_{k-1} & y_{k-1} & {\dot{y}}_{k-1} \end{array}\right]}^{T}$, respectively, we obtain the state vector of the target in the $x^{e}$ − $y^{e}$ Cartesian coordinate system, as:

(16) $$X_{k-1}^{e}=\left[\begin{array}{c} x_{k-1}^{e}\\ {\dot{x}}_{k-1}^{e}\\ y_{k-1}^{e}\\ {\dot{y}}_{k-1}^{e} \end{array}\right]=\left[\begin{array}{c} 0\\ \sqrt{{\dot{x}}_{k-1}^{2}+{\dot{y}}_{k-1}^{2}}\\ 0\\ 0 \end{array}\right]$$

Since the target moves at turn rate $\omega_{k}$, from time k−1 to time k, the state transition matrix of the target motion is given by:

(17) $$F(\omega_{k})=\left[\begin{array}{cccc} 1 & \frac{\sin\omega_{k}\Delta t_{k}}{\omega_{k}} & 0 & \frac{\cos\omega_{k}\Delta t_{k}-1}{\omega_{k}}\\ 0 & \cos\omega_{k}\Delta t_{k} & 0 & -\sin\omega_{k}\Delta t_{k}\\ 0 & \frac{1-\cos\omega_{k}\Delta t_{k}}{\omega_{k}} & 1 & \frac{\sin\omega_{k}\Delta t_{k}}{\omega_{k}}\\ 0 & \sin\omega_{k}\Delta t_{k} & 0 & \cos\omega_{k}\Delta t_{k} \end{array}\right]$$

where $\Delta t_{k}=t_{k}-t_{k-1}$ is the interval between times k and k−1. Using the state transition matrix in Equation (17), we obtain the state vector of the target in the $x^{e}$ − $y^{e}$ Cartesian coordinate system at time k, as:

(18) $$X_{k}^{e}=\left[\begin{array}{c} x_{k}^{e}\\ {\dot{x}}_{k}^{e}\\ y_{k}^{e}\\ {\dot{y}}_{k}^{e} \end{array}\right]=F(\omega_{k})X_{k-1}^{e}=\left[\begin{array}{c} \frac{\sqrt{{\dot{x}}_{k-1}^{2}+{\dot{y}}_{k-1}^{2}}\sin\omega_{k}\Delta t_{k}}{\omega_{k}}\\ \sqrt{{\dot{x}}_{k-1}^{2}+{\dot{y}}_{k-1}^{2}}\cos\omega_{k}\Delta t_{k}\\ \frac{\sqrt{{\dot{x}}_{k-1}^{2}+{\dot{y}}_{k-1}^{2}}(1-\cos\omega_{k}\Delta t_{k})}{\omega_{k}}\\ \sqrt{{\dot{x}}_{k-1}^{2}+{\dot{y}}_{k-1}^{2}}\sin\omega_{k}\Delta t_{k} \end{array}\right]$$

Based on Equation (18), the position coordinates of point E in the $x^{e}$ − $y^{e}$ Cartesian coordinate system are given by:

(19) $$\left[\begin{array}{c} x_{k}^{e}\\ y_{k}^{e} \end{array}\right]=\left[\begin{array}{c} \frac{\sqrt{{\dot{x}}_{k-1}^{2}+{\dot{y}}_{k-1}^{2}}\sin\omega_{k}\Delta t_{k}}{\omega_{k}}\\ \frac{\sqrt{{\dot{x}}_{k-1}^{2}+{\dot{y}}_{k-1}^{2}}(1-\cos\omega_{k}\Delta t_{k})}{\omega_{k}} \end{array}\right]$$

and the angle $\beta_{k}$ in Figure 2 is given by:

(20) $$\beta_{k}=arc\tan\frac{y_{k}^{e}}{x_{k}^{e}}=arc\tan\frac{1-\cos\omega_{k}\Delta t_{k}}{\sin\omega_{k}\Delta t_{k}}$$

We assume that a sensor observes the position of the target and we use ($r_{x}$, $r_{y}$) to denote the position measurement of the target in the x − y Cartesian coordinate system at time k. Obviously, the position measurement of the target at time k is its position coordinates at this time, if no measurement error appears in the measurement of the sensor. Therefore, we have:

(21) $$\left[\begin{array}{c} r_{x}\\ r_{y} \end{array}\right]=\left[\begin{array}{c} x_{k}\\ y_{k} \end{array}\right]\;\mathrm{or}\;\left[\begin{array}{c} r_{x}^{e}\\ r_{x}^{e} \end{array}\right]=\left[\begin{array}{c} x_{k}^{e}\\ y_{k}^{e} \end{array}\right]$$

where ($r_{x}^{e}$,$r_{y}^{e}$) is given by:

(22) $$\left[\begin{array}{c} r_{x}^{e}\\ r_{y}^{e} \end{array}\right]=\left[\begin{array}{cc} \cos\alpha_{k-1} & \sin\alpha_{k-1}\\ -\sin\alpha_{k-1} & \cos\alpha_{k-1} \end{array}\right]\times \left(\left[\begin{array}{c} r_{x}\\ r_{y} \end{array}\right]-\left[\begin{array}{c} x_{k-1}\\ y_{k-1} \end{array}\right]\right)$$

Replacing ($x_{k}^{e}$, $y_{k}^{e}$) in Equation (20) with ($r_{x}^{e}$, $r_{y}^{e}$) in Equation (22), we obtain:

(23) $$arc\tan\frac{r_{y}^{e}}{r_{x}^{e}}=arc\tan\frac{1-\cos\omega_{k}\Delta t_{k}}{\sin\omega_{k}\Delta t_{k}}$$

Let $c=\frac{r_{y}^{e}}{r_{x}^{e}}$ and $\phi =\omega_{k}\Delta t_{k}$, we have:

(24) $$(c^{2}+1)\cos^{2}\phi -2\cos\phi -c^{2}+1=0$$

Solving Equation (24), we obtain:

(25) $$\omega_{k}=\left\{ \begin{array}{l} \frac{1}{\Delta t_{k}}arc\cos\frac{{\left(r_{x}^{e}\right)}^{2}-{\left(r_{y}^{e}\right)}^{2}}{{\left(r_{x}^{e}\right)}^{2}+{\left(r_{y}^{e}\right)}^{2}},\;if\;r_{y}^{e}\geq 0\\ -\frac{1}{\Delta t_{k}}arc\cos\frac{{\left(r_{x}^{e}\right)}^{2}-{\left(r_{y}^{e}\right)}^{2}}{{\left(r_{x}^{e}\right)}^{2}+{\left(r_{y}^{e}\right)}^{2}},\;if\;r_{y}^{e}<0 \end{array}\right.$$

where:

(26) $$\left\{ \begin{array}{l} r_{x}^{e}=(r_{x}-x_{k-1})\cos\alpha_{k-1}+(r_{y}-y_{k-1})\sin\alpha_{k-1}\\ r_{y}^{e}=-(r_{x}-x_{k-1})\sin\alpha_{k-1}+(r_{y}-y_{k-1})\cos\alpha_{k-1} \end{array}\right.$$

Obviously, if no measurement error appears in the position measurement of the target, $\omega_{k}$ in Equation (25) is its turn rate from time k−1 to time k. Otherwise, we use $\omega_{k}$ in Equation (25) as the estimation of its turn rate from time k−1 to time k. Thus, by using the state vector of a target at a previous time, and its position measurement at the current time, we may estimate its turn rate at the current time.

## 4. MB Filter with an Adaptive Estimation of Turn Rate

In [19,20], Liu presented the MB filter to track multiple targets in the presence of clutter and noise. In this section, we apply the estimation method of the turn rate to the MB filter in [20], in order to develop the MB filter with an adaptive estimation of the turn rate. This filter consists of the following four steps:

### Step 1: Prediction

In this step, we predict the marginal distribution and existence probability of each target at the current time, according to its marginal distribution, existence probability, and the estimation of the turn rate at a previous time.

Let $N(x_{i,k-1};m_{i,k-1},P_{i,k-1})$, $\rho_{i,k-1}$, and $\omega_{i,k-1}$ denote the marginal distribution of target i, its existence probability, and the estimation of the turn rate at time k−1, respectively, where $i=1,2,\cdots ,N_{k-1}$ $N_{k-1}$ is the target number and N(⋅;m,P) is a Gaussian distribution with mean vector m and covariance matrix P. The predicted marginal distribution, existence probability, and turn rate of each target at time k are given by:

(27) $$N(x_{i,k};m_{i,k|k-1},P_{i,k|k-1}),i=1,2,\cdots ,N_{k-1}$$

(28) $$\rho_{i,k|k-1}=p_{S,k}\rho_{i,k-1},i=1,2,\cdots ,N_{k-1}$$

(29) $$\omega_{i,k|k-1}=\omega_{i,k-1},i=1,2,\cdots ,N_{k-1}$$

where $p_{S,k}$ is the survival probability, and $m_{i,k|k-1}$ and $P_{i,k|k-1}$ are given by:

(30) $$m_{i,k|k-1}=F(\omega_{i,k-1})m_{i,k-1}$$

(31) $$P_{i,k|k-1}=Q_{i,k-1}+F(\omega_{i,k-1})P_{i,k-1}F^{T}(\omega_{i,k-1})$$

where *T* denotes the transpose, $Q_{i,k-1}$ is the covariance of process noise, and $F(\omega_{i,k-1})$ is given by:

(32) $$F(\omega_{i,k-1})=\left[\begin{array}{cccc} 1 & \frac{\sin(\omega_{i,k-1}\Delta t_{k})}{\omega_{i,k-1}} & 0 & -\frac{1-\cos(\omega_{i,k-1}\Delta t_{k})}{\omega_{i,k-1}}\\ 0 & \cos(\omega_{i,k-1}\Delta t_{k}) & 0 & -\sin(\omega_{i,k-1}\Delta t_{k})\\ 0 & \frac{1-\cos(\omega_{i,k-1}\Delta t_{k})}{\omega_{i,k-1}} & 1 & \frac{\sin(\omega_{i,k-1}\Delta t_{k})}{\omega_{i,k-1}}\\ 0 & \sin(\omega_{i,k-1}\Delta t_{k}) & 0 & \cos(\omega_{i,k-1}\Delta t_{k}) \end{array}\right]$$

### Step 2: Estimation of Turn Rate

In this step, we use the measurements at time k, and marginal distribution of target i at time k−1, to estimate its turn rate from time k−1 to time k.

Let $y_{j,k}={\left[\begin{array}{cc} r_{x,k}^{j} & r_{y,k}^{j} \end{array}\right]}^{T}$ denote the measurement at time k, where $j=1,2,\cdots ,M_{k}$, $M_{k}$ is the number of measurements, and $r_{x,k}^{j}$ and $r_{y,k}^{j}$ denote the x and y components of measurement $y_{j,k}$; and let $m_{i,k-1}={\left[\begin{array}{cccc} \eta_{x,k-1}^{i} & {\dot{\eta }}_{x,k-1}^{i} & \eta_{y,k-1}^{i} & {\dot{\eta }}_{y,k-1}^{i} \end{array}\right]}^{T}$ denote the state vector of target i at time k−1, where $\left(\eta_{x,k-1}^{i},\eta_{y,k-1}^{i}\right)$ denotes the position coordinates of target i and $\left({\dot{\eta }}_{x,k-1}^{i},{\dot{\eta }}_{y,k-1}^{i}\right)$ denotes its velocities. According to Equation (25), the turn rate of target i that corresponds with measurement $y_{j,k}$, is given by:

(33) $$\omega_{k,e}^{i,j}=\frac{\mathrm{sgn}(y_{i,j}^{e})}{t_{k}-t_{k-1}}\arccos\frac{{\left(x_{i,j}^{e}\right)}^{2}-{\left(y_{i,j}^{e}\right)}^{2}}{{\left(x_{i,j}^{e}\right)}^{2}+{\left(y_{i,j}^{e}\right)}^{2}},i=1,2,\cdots ,N_{k-1},j=1,2,\cdots ,M_{k}$$

where

(34) $$\left[\begin{array}{c} x_{i,j}^{e}\\ y_{i,j}^{e} \end{array}\right]=\left[\begin{array}{cc} \cos\alpha_{i,k-1} & \sin\alpha_{i,k-1}\\ -\sin\alpha_{i,k-1} & \cos\alpha_{i,k-1} \end{array}\right]\times \left(\left[\begin{array}{c} r_{x,k}^{j}\\ r_{y,k}^{j} \end{array}\right]-\left[\begin{array}{c} \eta_{x,k-1}^{i}\\ \eta_{y,k-1}^{i} \end{array}\right]\right)$$

(35) $$\alpha_{i,k-1}=arc\cos\frac{{\dot{\eta }}_{x,k-1}^{i}}{\sqrt{{\left({\dot{\eta }}_{x,k-1}^{i}\right)}^{2}+{\left({\dot{\eta }}_{y,k-1}^{i}\right)}^{2}}}$$

(36) $$\mathrm{sgn}(y_{i,j}^{e})=\left\{ \begin{array}{ll} 1, & if\;y_{i,j}^{e}\geq 0\\ -1, & if\;y_{i,j}^{e}<0 \end{array}\right.$$

Considering that the turn rate of a target is generally within a known range, the turn rate of target i that corresponds with measurement $y_{j,k}$, may be given by:

(37) $$\omega_{k}^{i,j}=\left\{ \begin{array}{ll} \omega_{\max} & if\;\omega_{k,e}^{i,j}\geq \omega_{\max}\\ \omega_{k,e}^{i,j} & if\;-\omega_{\max}<\omega_{k,e}^{i,j}<\omega_{\max}\\ -\omega_{\max} & if\;\omega_{k,e}^{i,j}\leq -\omega_{\max} \end{array}\right.$$

where $\omega_{\max}$ is the maximal turn rate.

### Step 3: Update

In this step, we use the marginal distribution $N(x_{i,k-1};m_{i,k-1},P_{i,k-1})$, predicted existence probability $\rho_{i,k|k-1}$, turn rate estimation $\omega_{k}^{i,j}$, and measurement $y_{j,k}$, to obtain the updated marginal distribution, existence probability, and turn rate. The updated distribution and existence probability of target i that corresponds with measurement $y_{j,k}$, are as follows:

(38) $$N(x_{i,k};m_{k}^{i,j},P_{k}^{i,j}),i=1,2,\cdots ,N_{k-1},j=1,2,\cdots ,M_{k}$$

(39) $$\rho_{k}^{i,j}=\frac{p_{D,k}\rho_{i,k|k-1}N(y_{j,k};H_{k}m_{k|k-1}^{i,j},H_{k}P_{k|k-1}^{i,j}H_{k}^{T}+R_{k})}{\lambda_{c,k}+p_{D,k}\sum_{e=1}^{N_{k-1}}\rho_{e,k|k-1}N(y_{j,k};H_{k}m_{k|k-1}^{e,j},H_{k}P_{k|k-1}^{e,j}H_{k}^{T}+R_{k})},i=1,2,\cdots ,N_{k-1},j=1,2,\cdots ,M_{k}$$

where:

(40) $$m_{k|k-1}^{i,j}=F(\omega_{k}^{i,j})m_{i,k-1}$$

(41) $$P_{k|k-1}^{i,j}=Q_{i,k-1}+F(\omega_{k}^{i,j})P_{i,k-1}F^{T}(\omega_{k}^{i,j})$$

(42) $$m_{k}^{i,j}=m_{k|k-1}^{i,j}+A_{i,j}\cdot (y_{j,k}-H_{k}m_{k|k-1}^{i,j})$$

(43) $$P_{k}^{i,j}=(I-A_{i,j}\cdot H_{k})P_{k|k-1}^{i,j}$$

(44) $$A_{i,j}=P_{k|k-1}^{i,j}H_{k}^{T}{\left[H_{k}P_{k|k-1}^{i,j}H_{k}^{T}+R_{k}\right]}^{-1}$$

where $H_{k}$ and $R_{k}$ are the observation matrix and covariance matrix of the observation noise, respectively, I denotes the identity matrix, $\lambda_{c,k}$ denotes the clutter rate, and $p_{D,k}$ denotes the detection probability.

Equations (27) and (38) indicate that a marginal distribution $N(x_{i,k-1};m_{i,k-1},P_{i,k-1})$ at time k−1 generates a prediction distribution $N(x_{i,k};m_{i,k|k-1},P_{i,k|k-1})$ and $M_{k}$ update distributions $N(x_{i,k};m_{k}^{i,j},P_{k}^{i,j})$, $j=1,2,\cdots ,M_{k}$ at time k. To decrease the computational load, we merge these $M_{k}+1$ distributions to a singular distribution. The merging procedures are as follows:

Let $N(x_{i,k};m_{k}^{i,j},P_{k}^{i,j})$, $j=1,2,\cdots ,M_{k}+1$ denote the $M_{k}+1$ distributions where $N(x_{i,k};m_{k}^{i,M_{k}+1},P_{k}^{i,M_{k}+1})=N(x_{i,k};m_{i,k|k-1},P_{i,k|k-1})$. Similarly, let $\rho_{k}^{i,j}$, $j=1,2,\cdots ,M_{k}+1$ and $\omega_{k}^{i,j}$, $j=1,2,\cdots ,M_{k}+1$ denote the corresponding existence probabilities and turn rates, respectively, where $\rho_{k}^{i,M_{k}+1}=\rho_{i,k|k-1}$ and $\omega_{k}^{i,M_{k}+1}=\omega_{i,k|k-1}$. We first find the index of the maximal existence probability from $\rho_{k}^{i,j}$, $j=1,2,\cdots ,M_{k}+1$, namely:

(45) $$q=\arg\underset{j\in \{1,\cdots ,M_{k}+1\}}{\max}\{\rho_{k}^{i,j}\}$$

We then use the distribution, existence probability, and turn rate with index q, as the marginal distribution of target i, its existence probability, and the turn rate at time k, respectively, namely:

(46) $$N(x_{i,k};m_{i,k},P_{i,k})=N(x_{i,k};m_{k}^{i,q},P_{k}^{i,q}),i=1,2,\cdots ,N_{k-1}$$

(47) $$\rho_{i,k}=\rho_{k}^{i,q},i=1,2,\cdots ,N_{k-1}$$

(48) $$\omega_{i,k}=\omega_{k}^{i,q},i=1,2,\cdots ,N_{k-1}$$

### Step 4: Generation of New Target Distribution and the Output of the Filter

In this step, we use the measurement at time k to generate the marginal distribution of the new target as:

(49) $$N(x_{j,k};m_{\gamma ,k}^{j},P_{\gamma ,k}^{j}),j=1,2,\cdots ,M_{k}$$

where $m_{\gamma ,k}^{j}$ is from measurement $y_{j,k}={\left[\begin{array}{cc} r_{x,k}^{j} & r_{y,k}^{j} \end{array}\right]}^{T}$, and $m_{\gamma ,k}^{j}={\left[\begin{array}{cccc} r_{x,k}^{j} & 0 & r_{y,k}^{j} & 0 \end{array}\right]}^{T}$ and $P_{\gamma ,k}^{j}=P_{\gamma }$ where $P_{\gamma }$ is a known covariance matrix. Meanwhile, we designate parameter $\rho_{\gamma }$ as the existence probability of the new target and assign 0 as its turn rate, namely:

(50) $$\rho_{\gamma ,k}^{j}=\rho_{\gamma },j=1,2,\cdots ,M_{k}$$

(51) $$\omega_{\gamma ,k}^{j}=0,j=1,2,\cdots ,M_{k}$$

We then combine the marginal distributions of the existing targets in Equation (46), with those of new targets in Equation (49), to form the marginal distributions of individual targets at time k, as:

(52) $${\left\{N(x_{i,k};m_{i,k},P_{i,k})\right\}}_{i=1}^{N_{k}}={\left\{N(x_{i,k};m_{i,k},P_{i,k})\right\}}_{i=1}^{N_{k-1}}\cup {\left\{N(x_{j,k};m_{\gamma ,k}^{j},P_{\gamma ,k}^{j})\right\}}_{j=1}^{M_{k}}$$

where $N_{k}=N_{k-1}+M_{k}$. The corresponding existence probabilities and turn rates of individual targets at time k, are as follows:

(53) $${\left\{\rho_{i,k}\right\}}_{i=1}^{N_{k}}={\left\{\rho_{i,k}\right\}}_{i=1}^{N_{k-1}}\cup {\left\{\rho_{\gamma ,k}^{j}\right\}}_{j=1}^{M_{k}}$$

(54) $${\left\{\omega_{i,k}\right\}}_{i=1}^{N_{k}}={\left\{\omega_{i,k}\right\}}_{i=1}^{N_{k-1}}\cup {\left\{\omega_{\gamma ,k}^{j}\right\}}_{j=1}^{M_{k}}$$

After this combination, we prune the targets whose existence probability $\rho_{i,k}$ is less than threshold τ, and propagate the marginal distributions, existence probabilities, and turn rates of the remaining targets to the next time step. Those targets whose existence probability $\rho_{i,k}$ is greater than 0.5 are picked as the output of the filter at time k.

## 5. Simulation Results

In this section, we use an example to reveal the tracking performance of the MB filter with an adaptive estimation of the turn rate for multiple maneuvering targets. In this example, Pasha’s PHD filter [16] is used as the comparison object, and the OSPA distance [21], with parameters c=50 m and p=2, is used as the measure. The covariance matrix $Q_{i,k-1}$, observation matrix $H_{k}$, and covariance matrices $R_{k}$ and $P_{\gamma }$ used in the experiment, are as follows:

(55) $$Q_{i,k-1}=Q(\sigma_{v})=\left[\begin{array}{cccc} \frac{\Delta t_{k}^{4}}{4} & \frac{\Delta t_{k}^{3}}{2} & 0 & 0\\ \frac{\Delta t_{k}^{3}}{2} & \Delta t_{k}^{2} & 0 & 0\\ 0 & 0 & \frac{\Delta t_{k}^{4}}{4} & \frac{\Delta t_{k}^{3}}{2}\\ 0 & 0_{k} & \frac{\Delta t_{k}^{3}}{2} & \Delta t_{k}^{2} \end{array}\right]\sigma_{v}^{2}$$

(56) $$H_{k}=\left[\begin{array}{cccc} 1 & 0 & 0 & 0\\ 0 & 0 & 1 & 0 \end{array}\right]$$

(57) $$R_{k}=\left[\begin{array}{cc} \sigma_{w}^{2} & 0\\ 0 & \sigma_{w}^{2} \end{array}\right]$$

(58) $$P_{\gamma }=\left[\begin{array}{cccc} 2500 & 0 & 0 & 0\\ 0 & 625 & 0 & 0\\ 0 & 0 & 2500 & 0\\ 0 & 0 & 0 & 625 \end{array}\right]$$

where $\sigma_{v}$ and $\sigma_{w}$ denote the standard deviations of noises.

Three coordinated turn models with different turn rates are used in Pasha’s PHD filter. The state transition and covariance matrices for models $r_{i,k}=1$, $r_{i,k}=2$, and $r_{i,k}=3$ are given by $F_{k-1}(r_{i,k}=1)=F(\omega_{k}=0^{\circ }s^{-1})$, $Q_{k-1}(r_{i,k}=1)=Q(\sigma_{v}=1\;{\mathrm{ms}}^{-2})$, $F_{k-1}(r_{i,k}=2)=F(\omega_{k}=5^{\circ }s^{-1})$, $Q_{k-1}(r_{i,k}=2)=Q(\sigma_{v}=3\;{\mathrm{ms}}^{-2})$, $F_{k-1}(r_{i,k}=3)=F(\omega_{k}=-5^{\circ }s^{-1})$ and $Q_{k-1}(r_{i,k}=3)=Q(\sigma_{v}=3\;{\mathrm{ms}}^{-2})$, respectively. The Markov transition probabilities among different motion models are given by:

(59) $$[t_{k|k-1}(r_{i,k}|r_{i,k-1})]=\left[\begin{array}{ccc} 0.9 & 0.05 & 0.05\\ 0.05 & 0.9 & 0.05\\ 0.05 & 0.05 & 0.9 \end{array}\right]$$

$H(r_{i,k})$ and $R(r_{i,k})$ of Pasha’s PHD filter are given by $H(r_{i,k})=H_{k}$ and $R(r_{i,k})=R_{k}$.

**Figure 3:**
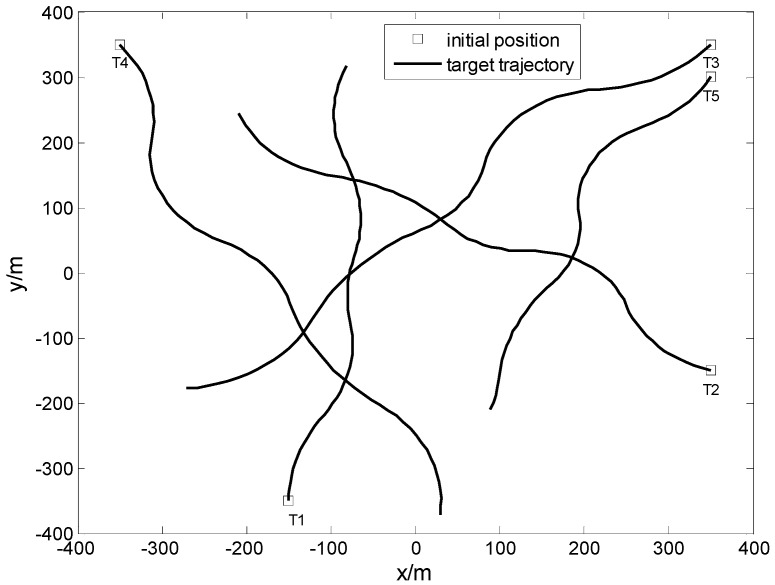
Moving trajectories of five targets.

**Example** **1.** Five targets are considered in this example. Targets 1, 2, 3, and 4 appear at t=1 s, t=1 s, t=3 s, and t=3 s, respectively, and disappear at t=70 s. Target 5 appears at t=5 s and disappears at t=60 s. Each target changes its turn rate at t=15 s, t=30 s, t=40 s, and t=55 s, respectively. The initial positions and moving trajectories of these five targets are shown in Figure 3.

**Figure 4:**
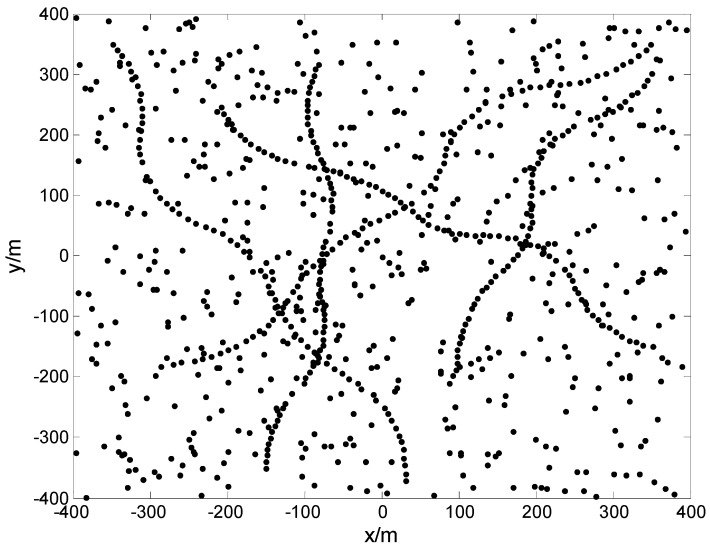
Simulation measurement for a trial in Example 2.

**Figure 5:**
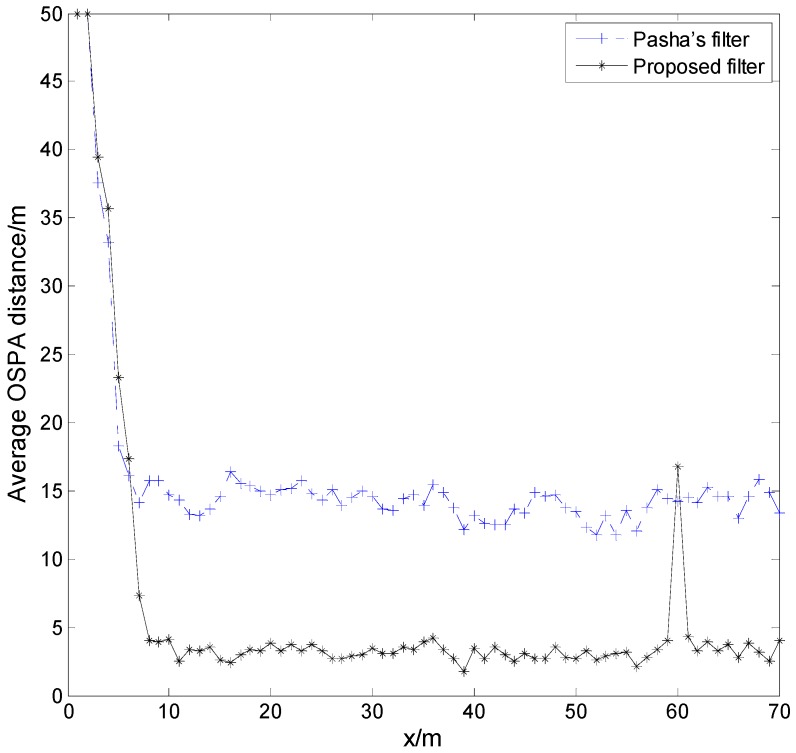
The experimental results for 150 trials.

We use parameters $\Delta t_{k}=1\;s$, $\sigma_{v}=0\;{m/s}^{2}$, $\sigma_{w}=1\;m$, $p_{S,k}=1.0$, $\lambda_{c,k}=1.25\times 10^{-5}\;m^{-2}$, and $p_{D,k}=0.95$ to generate the simulation measurement. Figure 4 shows the simulation measurement for a trial. Setting the parameters of the proposed filter to $\Delta t_{k}=1\;s$, $\sigma_{v}=1\;m/s$, $p_{S,k}=0.6$, $\lambda_{c,k}=1.25\times 10^{-5}\;m^{-2}$, $p_{D,k}=0.95$, τ=0.001, $\sigma_{w}=2\;m$, $\rho_{\gamma }=0.1$, and $\omega_{\max}=6{\;}^{o}/s$, and the parameters of Pasha’s filter to $\Delta t_{k}=1\;s$, $\sigma_{v}=1\;m/s$, $p_{S,k}=1.0$, $\lambda_{c,k}=1.25\times 10^{-5}\;m^{-2}$, $p_{D,k}=0.95$, τ=0.001, $\sigma_{w}=2\;m$, and $\rho_{\gamma }=0.1$, respectively, we use the proposed filter and Pasha’s filter to address the simulation measurements for 150 trials. The experimental results are shown in Figure 5. Based on these experimental results, the proposed filter performs better than Pasha’s filter, most of the time. Two factors are responsible for this result. The first factor is the difference between the actual motion mode of a target, and the dynamic model used by the filter. This difference causes Pasha’s filter to provide an inaccurate state estimation of the target. The proposed filter reduces this difference by estimating the turn rate of the target at each given time. The second factor is the filter’s memory. Due to the poor memory of Pasha’s filter, it is prone to discarding the information of a target from the posterior intensity, and cannot provide its state estimation if the target is not detected by a sensor at each point in time. In contrast to Pasha’s filter, the proposed filter provides the state estimation of a missed target, due to its sufficient memory of the target. The effect of the filter’s memory on the OSPA distance has been discussed in detail in [20]. As shown in Figure 5, a peak appears at t=60 s, because the proposed filter furnishes the state estimation of target 5 at its disappearing time. According to the definition of OSPA distance in [21], the OSPA distance is used to measure the similarity between two different sets. The excessive or deficient state estimation of the target will be punished with the cutoff distance.

To reveal the effect of the clutter rate and detection probability on the tracking performance of the proposed filter, we use different clutter rates and detection probabilities to generate simulation measurements, and use the proposed filter and Pasha’s filter to address the simulation measurements, respectively, for 150 trials. Table 1 and Table 2 show the results obtained from different clutter rates and detection probabilities. Table 1 suggests that an increase in the clutter rate leads to a larger OSPA distance for both the proposed filter and Pasha’s filter, but the proposed filter performs better at each clutter rate than Pasha’s filter. A similar conclusion is also reached from the result in Table 2. A decrease in the detection probability enlarges the OSPA distance of the proposed filter and Pasha’s filter, but the proposed filter obtains a smaller OSPA distance than Pasha’s filter at each detection probability.

The performance time is also an important measure for the performance of a filter. Table 3 displays the required time of a trial for the proposed filter and Pasha’s filter, at different clutter rates. Based on Table 3, the proposed filter requires more time than Pasha’s filter for each trial, because the proposed filter is used to estimate the turn rate of the target at each time step, and this estimation requires a number of calculations.

## 6. Conclusions

In this study, the formula for calculating the turn rate of a maneuvering target is derived. Based on this formula, we may estimate the turn rate of a target by using its state vector at a previous time, and its measurement at the current time. Applying the estimation method of the turn rate to the MB filter, we present a MB filter with an adaptive estimation of the turn rate, to track multiple targets maneuvering at a random turn rate. Based on simulation experimental data, we test the performance of the proposed filter, by comparing it with Pasha’s filter. The experimental results suggest that the proposed filter is better than Pasha’s filter at tracking the targets that maneuver at a variable turn rate.

## Figures and Tables

**Table 1:** OSPA distance (m) at five clutter rates.

$\lambda_{c,k}$ ($\times 10^{-6}\;m^{-2}$)	3.125	6.25	9.375	12.5	15.63
Proposed filter	5.18	5.61	6.05	6.32	6.48
Pasha’s filter	13.03	14.37	15.22	15.97	16.61

**Table 2:** OSPA distance (m) at five detection probabilities.

$p_{D,k}$	1.0	0.95	0.9	0.85	0.8
Proposed filter	5.04	6.32	7.79	11.67	12.95
Pasha’s filter	8.78	15.97	21.65	26.14	30.40

**Table 3:** Performance time (s) at five clutter rates.

$\lambda_{c,k}$ ($\times 10^{-6}\;m^{-2}$)	3.125	6.25	9.375	12.5	15.63
Proposed filter	3.14	4.96	7.70	10.70	14.37
Pasha’s filter	1.08	1.98	3.67	6.33	10.49
